# Decoupling Geometry and Surface Chemistry in 3D-Printed ALD-Functionalized Porous Ceramic Channels

**DOI:** 10.1021/acsomega.6c02566

**Published:** 2026-06-29

**Authors:** Antoine E. Jimenez, Diego R. Gomes, Carina Hedrich, Manuel Brinker, Fortune Minna, Patrick Huber, Kaline P. Furlan

**Affiliations:** † 150232 Karlsruhe Institute of Technology, Institute for Applied Materials, Ceramic Materials and Technology, Haid-und-Neu Straße 7, Karlsruhe 76131, Germany; ‡ Electron Microscopy Unit, Institute of Advanced Ceramics, 38987 Hamburg University of Technology, Eißendorfer Straße 42, Hamburg 21073, Germany; § Institute for Materials and X-Ray Physics, Hamburg University of Technology, Denickestraße 15, Hamburg 21073, Germany; ∥ 59198 Covenant University, KM 10 Idiroko Road, Ota 112104, Nigeria

## Abstract

Capillary-driven transport is traditionally attributed to the static pore geometry and wettability. However, the time-dependent surface energy of metal oxides, amplified by the high surface area of porous media, remains a key yet underexplored aspect. This study introduces a novel manufacturing route capable of decoupling macroscopic geometry from surface chemistry by integrating additive manufacturing combined with colloidal assembly (AMCA) and functionalization by atomic layer deposition (ALD). This approach enables the fabrication of highly porous aluminum­(III) oxide (Al_2_O_3_) and titanium dioxide (TiO_2_) ceramic channels after thermal burn-out. Within these structures, the structural and chemical properties are tuned and investigated. Spontaneous imbibition experiments at 0, 6, and 24 h after burn-out reveal a transition from a classical Lucas–Washburn rise to a resistance-limited regime dominated by evaporation and viscous drag. Time-resolved contact-angle measurements revealed that both oxides become superhydrophilic after burn-out and undergo subsequent hydrophobic recovery. Despite TiO_2_ being intrinsically more hydrophilic, Al_2_O_3_ channels consistently exhibited faster imbibition rates and a higher liquid rise. This behavior is attributed to the rapid surface relaxation of Al_2_O_3_, which reduces contact-line friction and minimizes pinning at high-energy adsorption sites, thereby enhancing fluid uptake. Macroscopic geometrical variations in printed channels did not affect the imbibition height but scaled linearly with imbibed volume, confirming the successful decoupling of geometric and chemical transport factors. The excellent structural reproducibility of the AMCA-ALD method establishes it as a robust manufacturing platform for programmable capillary transport. This approach provides a general pathway to design porous ceramics with independently engineered geometries and surface chemistries for applications in microfluidics, diagnostics, and catalysis.

## Graphical abstract

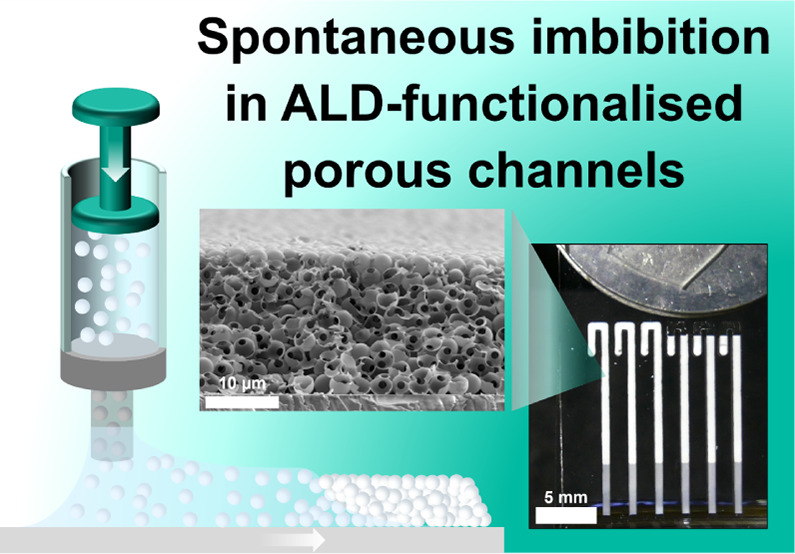

## 1 Introduction

Spontaneous imbibition is a capillary-driven transport mechanism that governs a wide range of natural and technological processes. In nature, it is responsible for water infiltration in plants as well as fluid transport at the cellular level.^1,2^ In technology, managing fluid transport in porous media is critical for various applications including energy storage, filtration, and catalysis.^3–5^ The efficiency of these processes relies on the interaction between fluids and the porous structure, which is determined not only by surface chemistry but also by pore geometry and interfacial properties.^6,7^

Top-down techniques like lithography are often used to fabricate porous structures capable of spontaneous imbibition.^8,9^ While precise, these methods limit material choice, involve long processing times, and significant waste generation due to etch-based process.^10^ Looking broadly into the fabrication methods for producing porous ceramics, other approaches include replica methods, sacrificial templating, and partial sintering.^11^ However, these methods often result in structures with pores’ sizes often on the upper range from millimeters to micrometers, thus presenting either limitations regarding the lower limit toward nanometric pore sizes or reduced control over the pore size distribution.

This work overcomes these limitations by 3D printing water-based polymeric suspensions by combining additive manufacturing with colloidal assembly (AMCA process).^12,13^ This approach enables the rapid fabrication of polymeric templates that are subsequently coated with ceramic layers using atomic layer deposition (ALD). After template burn-out, highly porous ceramic channels are obtained with uniform pore sizes, hereby named isoporous. In this work, aluminum­(III) oxide (Al_2_O_3_) and titanium dioxide (TiO_2_) were selected as coatings due to their distinct intrinsic wettability.^14,15^ A systematic investigation of spontaneous water imbibition in these channels was carried out (Figure 1). While the Lucas–Washburn model provides a basic framework for analyzing such dynamics, significant deviations are anticipated in these porous ceramic channels as dynamic wettability and evaporation become influential. The study demonstrates that fluid transport is not solely governed by the static contact angle, as predicted by some models. Instead, it is greatly influenced by the dynamic evolution of surface energy and its impact on contact line mobility.

**Figure 1:**
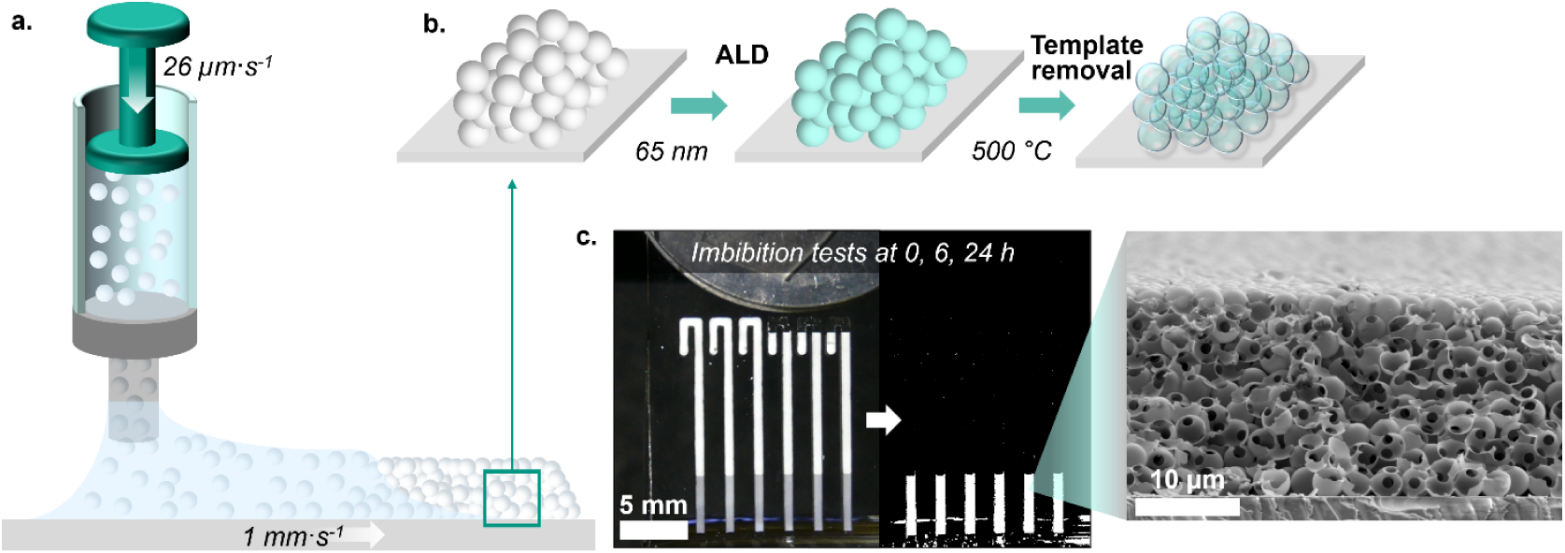
Technical workflow with parameters: (a) printing of colloidal templates, (b) conformal ALD coating and template removal, and (c) imbibition testing, illustrating the image processing (black-and-white conversion) used for water front tracking and a representative cross-section of a porous ceramic channel.

## 2 Materials and Methods

### 2.1 Additive Manufacturing Combined with Colloidal Assembly

Aqueous suspensions of polystyrene (PS) particles (Microparticles GmbH), with an average particle size of 2.94 ± 0.09 μm and a concentration of 100 mg·mL^–1^, were printed onto glass substrates (Polysciences Inc., Tissue Tack Microscope Slides, cat# 24216). Following a previous protocol developed by our group for the 3D printing of colloidal suspensions, the suspension concentration was increased to 300 mg·mL^–1^ prior to printing. The concentration was increased by pipet removal of the supernatant after 5 min centrifugation cycles (IKA mini-G, 6000 rpm).^13^ The concentrated suspensions were further homogenized in an ultrasonic bath for 15 min. The homogenized suspensions were then filled in 25 μL high-precision glass syringes (Hamilton Company, 1702 RN with 26s-gauge straight-type needle), which were mounted into a custom-made direct writing equipment. High-precision linear stages (Physics Instruments, M-126.2S1) defined the writing velocity of 1 mm·s^–1^ by controlling the substrate velocity in relation to the fixed needle (Figure 2a). Meanwhile, the plunger was actuated to extrude the suspension at a constant dispensing velocity of 26 μm·s^–1^. The printing process was monitored in situ by a camera (Edmund Optics, EO-10012C 1/2″ CMOS) and managed by executing macros written with GCS command (Physics Instruments, GCS Commands). In a typical printing run, the PS suspension is loaded in the syringe. A droplet is extruded at the needle tip with a fixed 0.2 mm plunger extrusion length as the substrate is raised to maintain a 100 μm gap between the needle tip and the substrate. The extruded droplet forms a liquid bridge upon contact with the substrate (Figure 2a). This interaction between the suspension and the substrate along the needle tip’s diameter creates a wet-pinned line as the substrate moves, ensuring a constant width during the process (Figure 2b).

**Figure 2:**
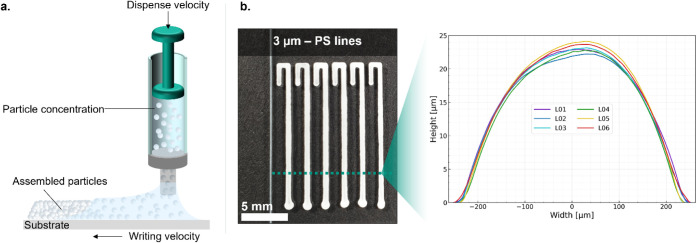
Overview of the printing process: (a) schematic of the AMCA process with a magnified view highlighting the colloidal assembly, (b) picture of printed template lines before burn-out with a line’s profilometry measurement. The plot displays six independent height profiles recorded from different lines on a single sample, illustrating the high reproducibility of the AMCA process.

### 2.2 ALD Functionalization and Template Removal

The printed PS lines (Figure 2b) were coated with 65 nm thick films of Al_2_O_3_ or TiO_2_ by ALD using a custom-made reactor (Hamburg University of Technology, TUHH). The Al_2_O_3_ film was deposited at a chamber temperature of 95 °C. For each cycle, the metal-organic precursor trimethylaluminum (Sigma-Aldrich, TMA) was introduced with a pulse/exposure/purge sequence of 0.06/15/90 s, subsequently followed by deionized water (0.12/15/90 s) to complete the surface reaction and form the oxide. In a similar procedure, TiO_2_ films were deposited using titanium­(IV) isopropoxide (Sigma-Aldrich, TTIP) heated to 85 °C, and introduced into the 95 °C chamber (1/30/90 s). Afterward, deionized water (0.2/30/90 s) was pulsed to form TiO_2_. The ALD reactor was constantly flushed with N_2_ as carrier gas at 2 L·h^–1^ during both deposition processes. Finally, the resulting films’ thicknesses of both materials were measured by ellipsometry (SENTECH, SENpro) on reference silicon wafers placed near the 3D-printed samples within the chamber.

After ALD, the coated lines were opened by manually removing their bottom section with a razor blade before the printed polymeric template was burned-out to obtain highly porous channels. The cut also assured an entrance opening for future water infiltration into the channels. The burn-out cycle was performed in air with a heating rate of 0.3 °C·min^–1^ from room temperature up to 500 °C, where the samples were kept for 30 min.

### 2.3 Characterization

The morphology of the printed lines was evaluated using optical profilometry (Alicona, Infinite Focus G4). Height profiles were measured across a 500 μm wide rectangular area at the beginning, middle, and end of each printed line to ensure representative profile results. The profiles’ sections were extracted from the raw data using a self-written Python script. Additionally, scanning electron microscopy (FEI, Nova NanoSEM 450) was used to verify the complete removal of the sacrificial template, as well as the pores’ arrangement.

The fluid transport properties of the channels were characterized using a custom-built imbibition setup (Figure S1) under controlled laboratory conditions (21.2 ± 0.7 °C and 21 ± 3% relative humidity). To ensure reproducible evaporation rates and minimize airflow fluctuations, all experiments were conducted in a still-air environment. For each experiment, a sample was mounted vertically on top of a Milli-Q (Merck Millipore, Milli-Q) water reservoir, located on a lifting platform. The capillary rise within a sample’s channels was recorded at 25 frames/s using a digital camera (Panasonic DMC-FZ300). To enhance the contrast between wet and dry regions, the sample was illuminated by a standard lamp (LED floodlight, 10 W). The recorded videos were further split into frames and analyzed from 0 to 100 s using custom Python scripts to extract the imbibition height automatically by tracking the evolution of the grayscale values. The initial position of the water meniscus in each channel was set as the zero-height reference to ensure that height measurements were independent of the initial meniscus shape.

Imbibition results were evaluated for both Al_2_O_3_ and TiO_2_ highly porous channels. For each material, the measurements were performed at three distinct elapsed times: immediately after PS burn-out and 6 or 24 h later. Each sample had up to eight printed lines to assess and up to three samples were analyzed for each material and time, respectively. This approach provided a robust data set of approximately 11 to 20 individual measurements per material and time interval, ensuring the repeatability of the observed imbibition. The resulting data are presented as mean values with error bars representing the standard deviation.

To aid on the evaluation of the acquired imbibition results, static contact angle measurements (dataphysics, OCA, Milli-Q water, *V*_droplet_ = 3 μL) were also conducted on the ALD-coated substrates (Figure S2).

## 3 Results and Discussion

### 3.1 Morphology of Printed Templates and Porous Ceramic Channels

The PS particles in the printed templates are arranged in a disordered manner, indicating no long-range order (Figure 3a,b). This disordered arrangement is characteristic of an evaporation-dominated self-assembly. In this work, rapid printing conditions promote this effect.^16^ The fast processing time of AMCA introduces several simultaneous drying phenomena, which makes defect suppression in micrometer-wide printed lines challenging.^17^ In this work, the printing parameters are optimized to hinder their formation. In addition to direct writing, the colloidal particles self-assembling during drying constitute the second component of the AMCA method.^12^ Traditional vertical convective self-assembly typically requires about 96 h to assemble 1 cm^2^. In contrast, the present direct-writing-based method produces the same area in roughly 10 min. Although conventional drop-casting can fabricate self-assembled structures in 20–30 min, it is prone to enlarged coffee-ring formation.^18,19^

**Figure 3:**
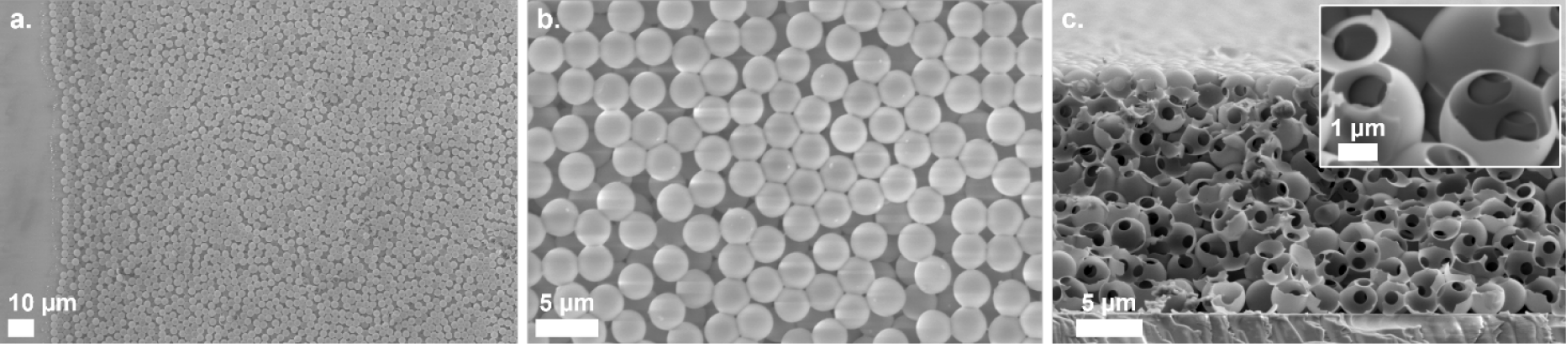
Scanning electron microscope images of a porous Al_2_O_3_ channel showing (a, b) top and (c) cross-sectional views.

Beyond the inherent speed of the direct-writing process, the AMCA-ALD platform has a significant potential for industrial scalability via parallelization. While the current study uses a single-nozzle configuration, the technique is adaptable to multinozzle printhead arrays. Furthermore, the thermal burn-out and ALD functionalization processes are batch compatible. Multiple samples have the same processing time as a single sample with the sole limitation being the dimensions of the furnace and the ALD chamber. As a result, throughput can be scaled effectively without increasing the total fabrication time. This combination of high-speed printing and batch processing makes the route highly suitable for large-scale production.

At the microscopic scale, particle mobility is governed by solvent evaporation.^20,21^ While the liquid bridge at the needle tip remains wet, the printed line begins to dry as the substrate is moved forward. Under standard laboratory conditions, drying preferentially occurs at the three-phase contact line between the substrate, suspension, and atmosphere.^18,22^ Fast printing induces fast drying, generating evaporation-driven capillary flows that transport particles toward the contact line. Given sufficient mobility, strong capillary flows drive particles to the edge, where they accumulate, giving rise to the coffee-ring effect. At a smaller scale, electrostatic interactions between particles and between particles and the substrate further influence their assembly.^21^ In this work, the coffee-ring effect is likely suppressed due to restricted particle mobility and surface-capturing phenomena, both promoted by the fast evaporation associated with the chosen printing parameters.^26,27^ These mechanisms are consistent with previous studies on colloidal self-assembly.^23,24^

Finally, the integrated AMCA-ALD platform (comprising template printing, conformal coating, and thermal burn-out) yields highly reproducible, crack-free ceramic channels with consistent morphology across independent batches. Average widths of 600 ± 64 μm and heights of 15.9 ± 3.3 μm show structural robustness, with the printed shape preserved after ALD coating and burn-out. This leads in highly uniform ceramic channels defined by the original colloidal packing (Figure 3c).

### 3.2 Imbibition Dynamics in ALD-Functionalized Porous Channels

Upon vertical contact with water, the liquid front spontaneously advances into the porous channels, showing spontaneous imbibition with a characteristic two-regime behavior (Figure 4). In the first few seconds, the water front moves rapidly and maintains a nearly flat profile across the channel’s width. As the imbibition rate decreases, the meniscus gradually deforms into a concave shape (Figure S3). The greater rise at the edges is attributed to the reduced local height of the printed semicylindrical channels. This phenomenon appears in all channels, independent of oxide type (Al_2_O_3_ or TiO_2_) or the time elapsed after burn-out (0, 6, or 24 h).

**Figure 4:**
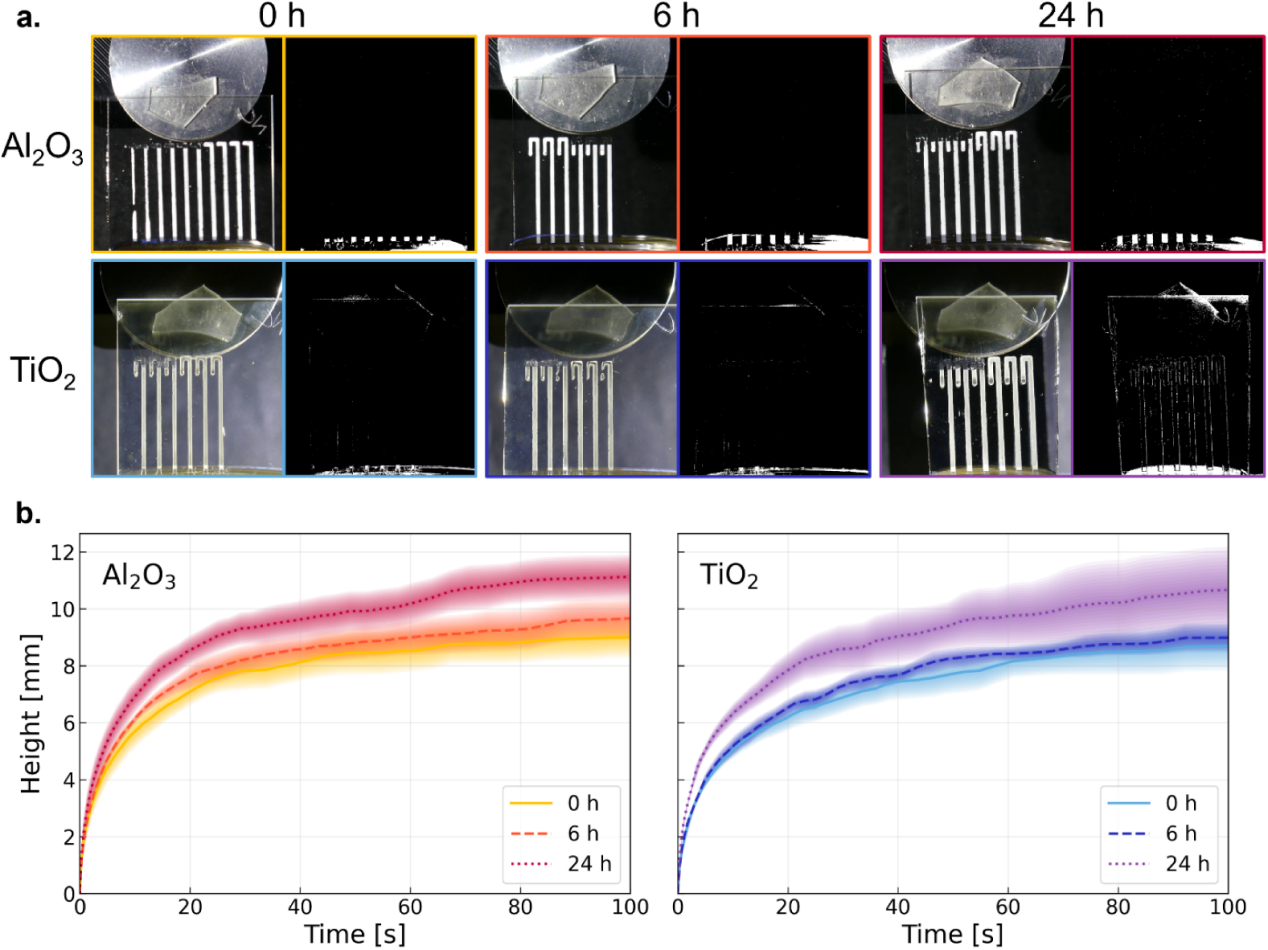
Imbibition results: (a) reference frames used as origins for each material and time, including the color conversion used for tracking water rise. Both open and closed channels can be identified on each sample. Scale is given by the 25 mm-diameter metallic stubs. (b) Imbibition height as a function of time for each material and after-burn-out time. Lines represent the mean values, while the shaded areas indicate the standard deviation (±1 SD).

The initial fast rise is followed by a shift of the imbibition rate toward a slower regime, in which the water front approaches a plateau. Such behavior is typical of spontaneous imbibition in porous media.^25,26^ During the early stage, capillary forces dominate, and the Lucas–Washburn model adequately describes the dynamics. The water front remains essentially flat because capillary forces overwhelm the viscous resistance and the disordered arrangement of pores averages the infiltration laterally. As the liquid progresses and viscous drag increases, the imbibition slows and the meniscus develops its concave profile.^27–29^

Plotting the imbibition height against the square root of time (Figure S4) confirms Lucas–Washburn behavior during the first few seconds, with all samples showing a linear relationship.^30,31^ Beyond this point, the measurements deviate from the classical model as viscous, gravitational, and evaporative contributions become non-negligible.^26,32,33^ In several samples, discrete height jumps are observed during the slower regime at different times. These jumps appear in both Al_2_O_3_ and TiO_2_, at all investigated sample types (0, 6, and 24 h), indicating that they are not experimental artifacts. Instead, they are consistent with Haines jumps: sudden advances of the liquid front when the meniscus overcomes a local pore constriction.^34^

To quantitatively analyze the imbibition behavior, Siebold’s parameter-extraction is combined with the macroscopic formulation of Fries and Dreyer.^35,36^ The detailed derivation and parameter extraction workflow are described in the Supporting Information (Section S2). In brief, the height evolution is described by the ordinary differential equation (ODE)

1 $$\frac{dh}{dt}=\frac{a}{h}-b-c\cdot h$$

where *a* contains the capillary driving pressure and viscous resistance, *b* accounts for gravity, and *c* represents the effect of evaporation; Introducing the complete ODE here provides a physical framework for interpreting the transition from the fast to slow imbibition regimes.

During the initial imbibition stage, gravity and evaporation are negligible, and the ODE reduces to $\frac{dh}{dt}\approx \frac{a}{h}$ . Thus, the slope of *h* ^*2*^(*t*) directly yields the coefficient *a*.

The Siebold analysis distinguishes between the static radius *R*_*S*_, which sets the capillary pressure, and the hydrodynamic radius *R*_*D*_, which controls viscous conductance. ALD-derived porous networks contain two characteristic throat sizes (Section S2b). These include smaller external three-sphere junction throats (Figure 3b, Figure S5a) and larger internal throats at former templating sphere–sphere contacts (Figure 3c, Figure S5b).^37^ As the driving capillary pressure is set by the smallest constriction, the external junction throat radius is taken as the effective *R*_*S*_. With *R*_*S*_ and *R*_*D*_ determined, the permeability *K* follows from Siebold’s relation. This allows the computation of the gravitational coefficient *b*. As for the evaporative coefficient *c*, it is obtained from the long-time plateau height using the steady-state form of the ODE. Sample-specific evaporative fluxes and all model fits are reported in the Supporting Information (Figure S6, Table S2).

Finally, the relative importance of gravity and evaporation becomes clear when the limiting heights are compared. In the hypothetical absence of evaporation (*c* = 0), the maximum height predicted by Jurin’s equation^26^ is about 1 order of magnitude higher than the experimentally observed rise. This confirms that, under the present conditions, evaporation rather than gravity sets the long-time limit of imbibition (Figure 5). This discrepancy reveals a fundamental physical difference in how these mechanisms scale. While gravity operates as a constant linear force, evaporation acts as a cumulative volumetric loss that increases proportionally with the wetted surface area. Consequently, as the water front advances and the total evaporative flux rises, a dynamic mass balance is reached, which determines the final plateau.^38^ This state, rather than a simple hydrostatic limit, causes the imbibition to stop when the rate of liquid loss in the channels matches the supply rate determined by the channel’s permeability.

**Figure 5:**
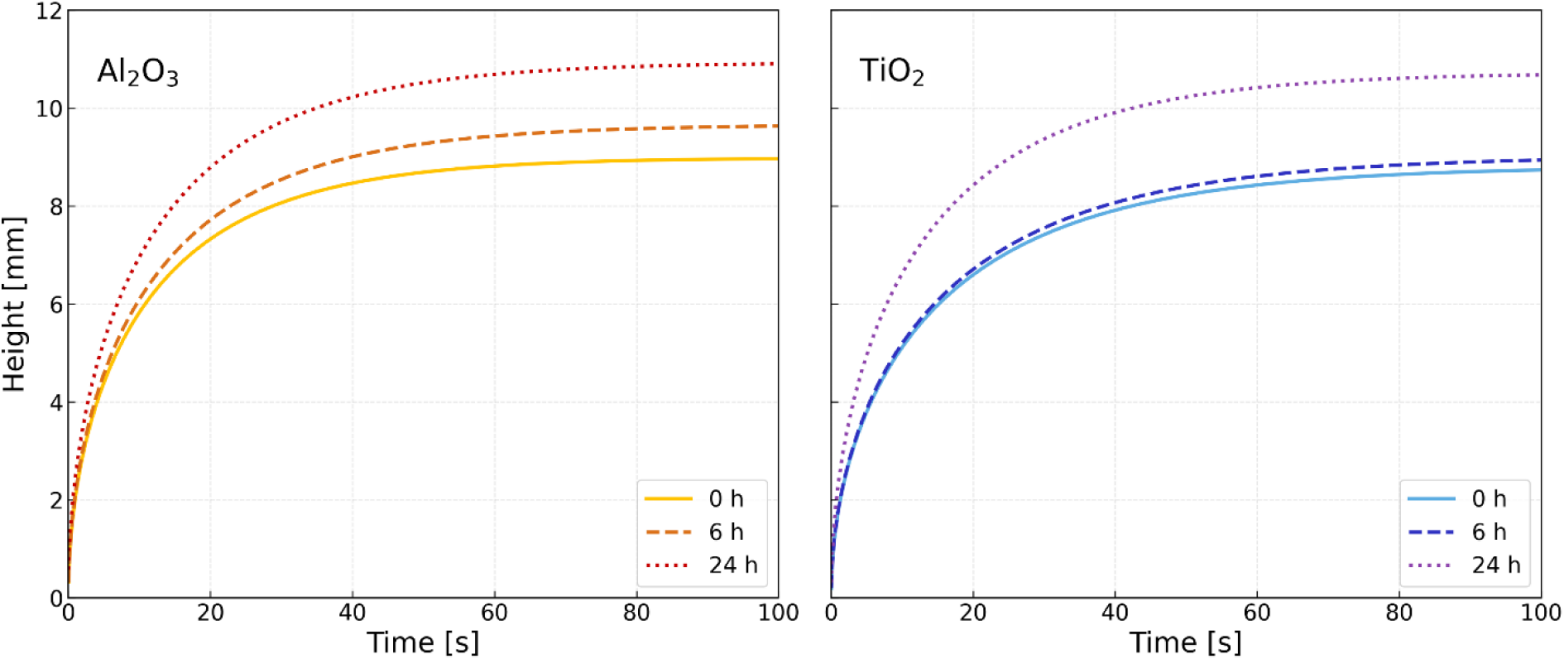
Fries–Dreyer’s ordinary differential equation fit of the imbibition dynamics.

To further investigate this evaporation contribution, closed (single opening at the bottom) and open (two openings at both ends) channels were fabricated within the same samples (Figure 4). In the time frame analyzed and despite comparable Lucas–Washburn slopes in the fast regime, closed Al_2_O_3_ channels consistently reach slightly higher heights than open ones immediately and 6 h after burn-out. After 24 h, both geometries exhibit similar imbibition performance. The average evaporative mass flux for all Al_2_O_3_ channels is 0.309 ± 0.080 g·m^–2^·s^–1^. This value exceeds typical flat-water values under still air. However, it is consistent with porous media where evaporation occurs at confined menisci. Additionally, large internal surface areas can increase local evaporative fluxes significantly.^39^ Open Al_2_O_3_ channels have a slightly higher evaporative flux than closed ones (Table S2), consistent with the additional opening facilitating vapor transport. The latter increased evaporation counteracts capillary rise, which explains why open Al_2_O_3_ channels do not achieve similar imbibition heights despite similar capillary driving forces.

In TiO_2_ samples, by contrast, closed and open geometries show almost no difference in either slope or final height at 0 and 6 h after burn-out. The average evaporative mass flux is 0.260 ± 0.050 g·m^–2^·s^–1^. This lower value is consistent with the more hydrophilic nature of TiO_2_ surfaces, where stronger liquid retention stabilizes the water meniscus and hinders evaporation (Section 3.3). Interestingly, after 24 h, open TiO_2_ channels exhibit a substantially higher imbibition height than closed ones, reversing the trend observed in Al_2_O_3_. This difference matches with the lower evaporative flux calculated in the open channels, hereby suggesting that the prolonged hydrophilicity of TiO_2_ effectively decreases the evaporation loss by stabilizing the liquid within the pores. Hence, the limiting factor in closed TiO_2_ channels is not only evaporation, but also the resistance from trapped air. As TiO_2_ retains its hydrophilicity after 24 h, the walls are more easily saturated with liquid, therefore hindering the escape of displaced air in the closed channel. The resulting backpressure likely slows the water front more significantly than the evaporative loss present in the open channels. Consequently, the open TiO_2_ channels have a better imbibition capability despite higher exposure to ambient air due to their remaining hydrophilicity. Detailed flux and height values for all materials and times are summarized in the Supporting Information (Table S2).

These observations demonstrate that channel openings do not influence the rapid imbibition in the first stage, where capillary forces dominate. Differences only become apparent in the slower regime, when evaporation and trapped air pressure start to compete with capillary-driven transport.^40,41^ Moreover, the time-dependent evolution of the open/closed behavior points to a contribution from surface chemistry changes. Thermal treatment at 500 °C and subsequent ambient humidity exposure are known to modify oxide surface energy and wettability through surface relaxation and adsorption processes.^42,43^ The interplay between this time-dependent surface chemistry and imbibition dynamics is examined in the next two sections.

### 3.3 Decoupling Surface Chemistry Effects on Imbibition

Static and dynamic wettability are two aspects of surface wetting. First, static wettability refers to the equilibrium of the system, represented by the static contact angle (θ), which determines the magnitude of the capillary force. In contrast, dynamic wettability describes the dissipative processes and resistance encountered by the advancing three-phase contact line, such as molecular pinning and contact-line friction. As demonstrated in the following analysis, these latter properties can evolve independently, leading to the observed transport paradox. The two ceramic materials, aluminum­(III) oxide and titanium dioxide, were chosen to create porous channels with comparable geometries but different surface chemistries. The static contact angle values provide a reference for the wettability of the corresponding ceramic channels. The measurements confirm that TiO_2_ is intrinsically more hydrophilic than Al_2_O_3_, with contact angles of θ = 40 ± 4° and θ = 71 ± 6°, respectively (Figure S2). According to the Lucas–Washburn equation, the imbibition rate is proportional to cos­(θ) (eq S1). Consequently, the more hydrophilic TiO_2_ channels are expected to exhibit faster and higher imbibition. Based on this equation, the ratio of expected heights should account to $h_{TiO_{2}}$ / $h_{Al_{2}O_{3}}$ = cos $\left(\theta_{{\mathrm{TiO}}_{2}}\right)$ /cos $\left(\theta_{Al_{2}O_{3}}\right)$ = 0.77/0.33 = 2.35, i.e., water should rise about 2.35 times higher in TiO_2_ channels.

Conversely, the experimental results show a consistent and unexpected trend. Across all time intervals after burn-out, Al_2_O_3_ channels demonstrate similar or greater imbibition performance. On average, the final height reached after 100 s is 4.66% greater in Al_2_O_3_ channels, while the spontaneous imbibition rate is 12.9% faster than in TiO_2_ (Table S1). Fluid transport in these channels is characterized by two distinct regimes. An initial rapid rise follows the Lucas–Washburn model (eq S1), while a subsequent slower phase is resistance-limited (eq 1). Materials analysis reveals a clear contradiction to the standard Lucas–Washburn expectations. Despite being intrinsically less hydrophilic, Al_2_O_3_ channels consistently outperform the more hydrophilic TiO_2_ channels. Furthermore, imbibition performance for both materials improves over time following burn-out. This trend is not limited to the final height reached after 100 s, where gravity and, more critically, evaporation effects dominate (Section 3.2). The same tendency is observed during the initial rise, which is expected to be a purely capillary-driven regime (Table S1). This demonstrates that the discrepancy is not caused solely by external resistances such as evaporation and gravity. Instead, it also originates from the effective parameters within the Lucas–Washburn equation itself (eq S1). It suggests that the effective wettability governing the capillary driving force is not a fixed intrinsic property but rather a dynamic parameter altered by the channel structure and by the thermal burn-out process.

Contact angle measurements performed at different times after burn-out (Figures S7 and S8) reveal that both oxides become superhydrophilic immediately after burn-out (time “0”), indicating a significant change in the surface chemistry, which agrees with reports by other authors.^15,42,43^ Water droplets spread out completely upon contact with the oxide films, yielding almost flat contact angles. Only Al_2_O_3_ tested 24 h after burn-out shows a measurable value (θ = 11 ± 1°), while TiO_2_ remained superhydrophilic. The loss of hydrophilicity, or hydrophobic recovery, over time has been reported previously and is attributed to surface relaxation and interactions with ambient humidity.^44^ For TiO_2_ substrates, contact angle relaxation occurs because maintaining hydrophilicity requires a critical density of donor–acceptor complexes between water molecules and surface hydroxyl groups (Ti–OH). When this threshold is not met, the surface relaxes and gradually recovers hydrophobicity.^45^ In this work, the faster hydrophobic recovery observed for ALD-coated Al_2_O_3_ suggests that fewer or less stable complexes are formed compared to TiO_2_. This indicates that the imbibition behavior in the produced porous channels is likely governed by time-dependent surface energetics rather than by static intrinsic hydrophilicity values.

The initial superhydrophilic behavior arises from the thermal removal of adsorbates and the creation of a high-energy oxide surface.^43,46,47^ The subsequent increase in contact angle is due to the aforementioned hydrophobic recovery, which gradually lowers the surface energy.^48^ In parallel, the imbibition capability of both materials improves with increasing time after burn-out. For instance, from 6 to 24 h, the final imbibition height increases by 12.7% for Al_2_O_3_ and 14.6% for TiO_2_. These results emphasize that imbibition in the metal oxide channels is not solely dictated by static wettability but is heavily impacted by dynamic wetting phenomena occurring during the capillary rise. While changes in surface chemistry modify both static and dynamic contact angles, their impact on fluid flow differs. On the one hand, the static contact angle sets the capillary driving force in the Lucas–Washburn context. On the other hand, dynamic wettability controls the contact-line mobility and the viscous dissipation occurring at the rising meniscus.^49–51^

These outcomes can be rationalized by considering dynamic wetting at the advancing three-phase contact line. In capillary-driven flows, the moving liquid front experiences drag due to molecular pinning.^52,53^ At the contact line, liquid molecules adsorb onto active sites of the solid surface. According to the molecular-kinetic theory of wetting, the movement of the contact line is viewed as a series of discrete molecular jumps between adsorption sites on the solid surface. Molecules must repeatedly overcome energy barriers to advance the contact line.^52–54^ On highly hydrophilic, high-energy surfaces, the density of such adsorption sites is high. While this high surface energy provides a strong static contribution (low static θ), it simultaneously increases the frequency of pinning events, leading to significant frictional losses and molecular drag.^50,52^ Consequently, a paradox arises: the surface with the strongest static driving force (TiO_2_) also possesses the highest dynamic resistance (Figure 6).

**Figure 6:**
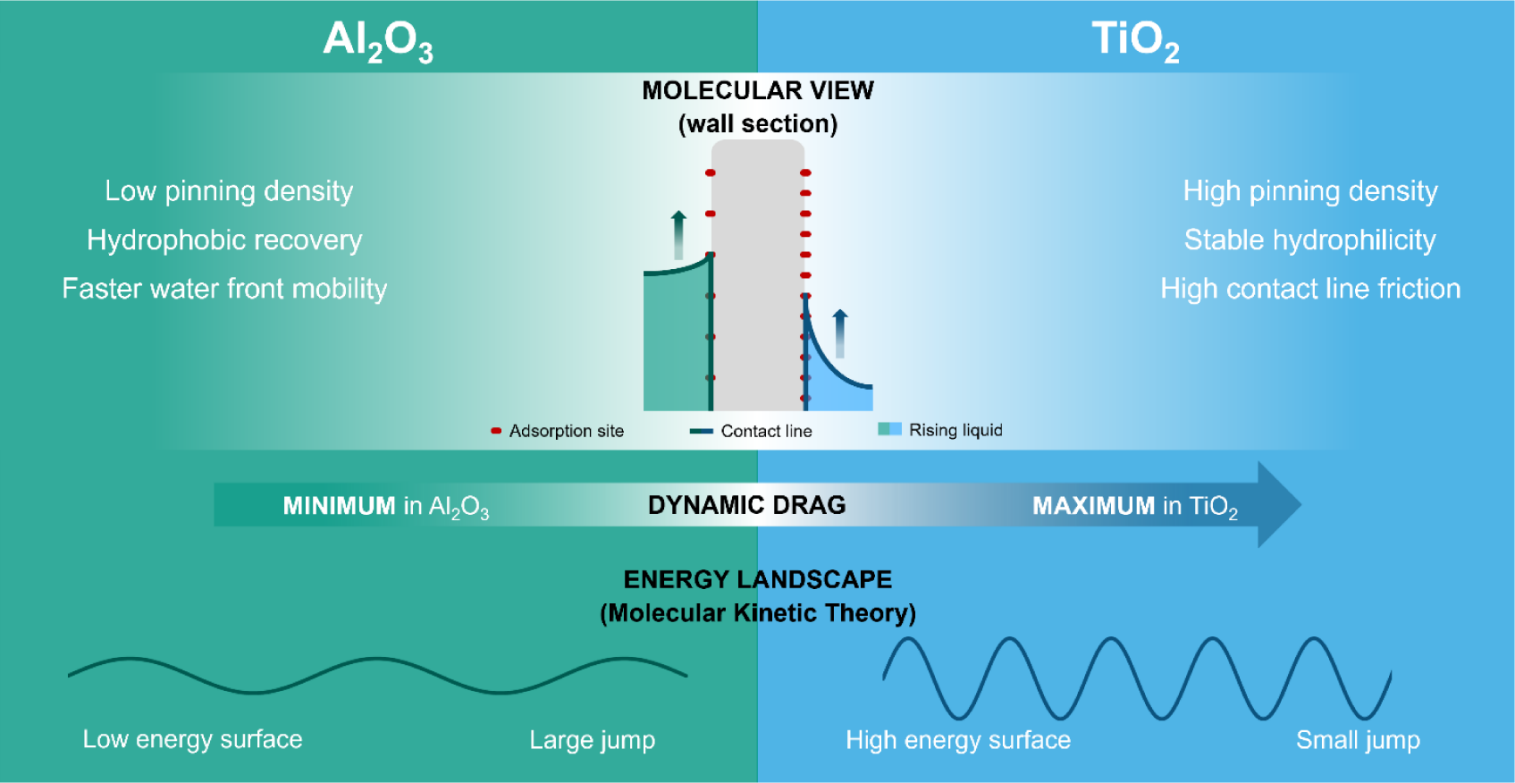
Conceptual mechanisms of the hydrophilic paradox. (Top) Molecular view of the rising contact line: TiO_2_ exhibits stable superhydrophilicity, but higher pinning density compared to the relaxed Al_2_O_3_ surface. (Bottom) Corresponding energy landscapes based on the Molecular Kinetic Theory.^52,55^

Over time, surface relaxation reduces the density of active sites and resulting in pinning. Thereby, it lowers contact-line resistance and enhances the liquid rise. Importantly, this resistance is not constant. As the surface ages, molecules from the ambient atmosphere adsorb onto the surface hydroxyl groups. Therefore, the number of hydroxyl groups available for molecular pinning decreases. With fewer active sites, the surface gradually loses its initial superhydrophilicity, and contact angle relaxation occurs, as discussed above. In line with the molecular-kinetic theory of wetting, the reduction in pinning decreases the resistance at the contact line and thereby increases the mobility of the liquid front.^53^

This time-dependent reduction in molecular drag appears to be the dominant effect in this work. It can even compensate for the gradual reduction of the capillary driving force associated with the increasing static contact angle. The consistently greater performance of Al_2_O_3_ channels compared to TiO_2_ can thus be explained by differences in their surface chemistry.^45,48^ Both Al_2_O_3_ and TiO_2_ hydroxylate, but hydroxyl groups on Al_2_O_3_ more readily relax upon exposure to ambient humidity, leading to faster hydrophobic recovery and reduced molecular pinning.^42,48,56^ TiO_2_, in contrast, maintains a higher density of strongly bound Ti–OH groups, which tend to form donor–acceptor complexes with water molecules.^45^ This stabilizes long-lasting hydrophilicity but also enhances pinning at the contact line, thereby hindering the mobility of the liquid front. In other words, weaker hydroxyl binding and lower surface energy in Al_2_O_3_ promote more efficient dynamic contact-line relaxation in the channels. In contrast, stronger hydroxyl-water interactions on TiO_2_ trap the contact line more effectively. Ultimately, these differences in dynamic surface energetics provide a rational explanation for the better imbibition performance observed in Al_2_O_3_ channels, despite TiO_2_ being intrinsically more hydrophilic.

### 3.4 Geometric Factors Influence

Geometric factors influence capillary-driven transport across a number of length scales. At the pore scale, the imbibition rate and rise height are regulated by the pores, which control capillary pressure and viscous resistance (Section 3.2). At the channel scale, the total volume of fluid uptake is expected to be proportional to the cross-sectional area, as is the case for simple cylindrical tubes.^26,57^ The pore radius and throat geometry in the printed metal oxide porous channels studied here are set by the template size and the self-assembly process and therefore remain constant across all samples. In contrast, the macroscopic channel geometry is fixed during printing and can vary slightly among samples.

The average channel width and height are 600 ± 64 μm and 15.9 ± 3.3 μm, respectively, indicating minimal variation for a direct-writing process. The mean cross-sectional area, obtained by integration of the measured profiles, is 6666 ± 785 μm^2^. Although this geometric variability is modest, it is sufficient to assess whether variations in channel-scale geometry affect the imbibition dynamics.

No significant correlation is found between the final imbibition height at 100 s and the cross-sectional area under any of the conditions studied (Figure 7a). This indicates that within the explored range, the imbibition capability is effectively independent of the overall cross-sectional width of the channel. This observation is consistent with the capillary theory, in which the rise height is primarily determined by pore-scale parameters (pore radius, liquid properties, and wettability), rather than by the macroscopic channel dimensions. In addition, the relatively small variation in cross-section may not be sufficient to induce substantial differences in hydraulic resistance or evaporation losses and thus in rise height. Width-dependent effects are typically reported only when the dimensions vary more significantly, with larger geometries sometimes showing higher rise due to reduced boundary effects.^56^

**Figure 7:**
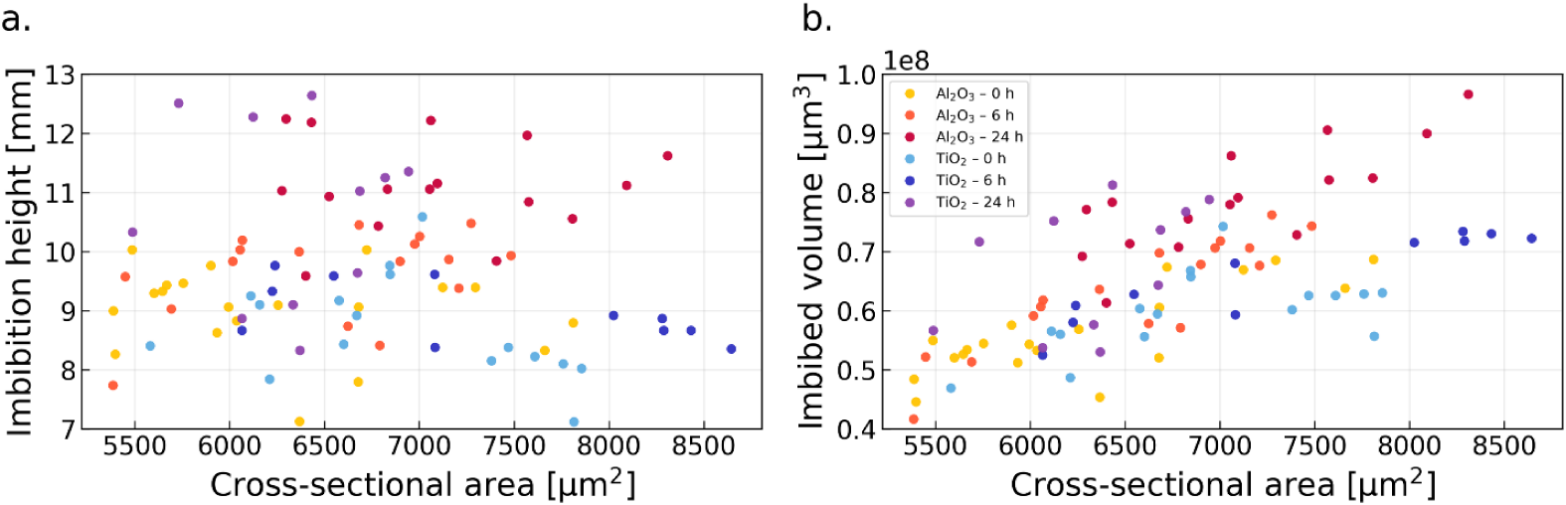
Correlations between water imbibition and channel geometry: (a) imbibition height at *t* = 100 s [mm] and (b) total imbibed volume [μm^3^], both plotted as functions of channels cross-sectional area [μm^2^]. Individual data points represent measurement from distinct channels across multiple samples.

In contrast, clear trends emerge when considering the total imbibed volume, calculated as the product of the imbibition height and cross-sectional area (Figure 7b). For both materials, the imbibed volume increases with the cross-sectional area, as expected. For Al_2_O_3_ channels, good Pearson correlations are found at 6 h (*r* = 0.79, *p* < 0.001) and 24 h (*r* = 0.59, *p* = 0.015). Similarly, TiO_2_ channels exhibit correlations at 0 h (*r* = 0.59, *p* = 0.012) and 24 h (*r* = 0.89, *p* < 0.001). In other words, imbibition height depends mainly on surface chemistry and dynamic wetting. However, the imbibed volume scales linearly with channels’ cross-section, as expected from geometric considerations.

This behavior distinguishes between bulk-like flow within the pore space and interfacial dynamics. At the pore walls, liquid–solid interactions introduce additional friction and control the rise dynamics.^25^ In other words, geometry dictates the uptake capacity, whereas the rise dynamics are mostly governed by pore-scale properties and surface chemistry.

Finally, the results confirm that fluid transport in the disordered porous channels is predominantly influenced by liquid–solid interactions and time-dependent surface chemistry, rather than macroscopic geometric features. As expected, channels’ cross-sections nonetheless determine the total volume of liquid uptake. Therefore, while macroscopic geometry does not influence the imbibition capability, it remains a critical design parameter for applications requiring maximized transport capacity.

## 4 Conclusion

In this work, Al_2_O_3_ and TiO_2_ isoporous channels were fabricated by combining the direct writing of colloidal suspensions (AMCA) with conformal ALD coatings. The method produces ceramic channels with well-defined pore size and reproducible line geometries, while allowing surface chemistry to be functionalized independently via the choice of ALD oxide.

Spontaneous water imbibition in these channels showed a robust two-stage behavior: an initial Lucas-Washburn regime followed by a resistance-limited regime dominated by evaporation. Within this framework, a key and counterintuitive result was that Al_2_O_3_ channels imbibe faster and higher than TiO_2_ channels despite the latter being intrinsically more hydrophilic on flat films. These findings established time-dependent surface energetics and contact-line friction as decisive factors, indicating that dynamic wetting can outweigh nominal static hydrophilicity in governing the capillary rise.

Meanwhile, geometrical analysis revealed that across the range of printed channel dimensions investigated here, the macroscopic cross-section did not affect water rise but linearly scaled the imbibed volume. This decoupling of rise dynamics (set by pore-scale properties and surface chemistry) from uptake capacity (set by the printed geometry) is an important design feature of the AMCA-ALD platform. Furthermore, the structural reproducibility and time efficiency of the method establish it as a robust manufacturing platform. The compatibility with parallelized printing via multinozzle arrays, as well as the batch-processing nature of thermal treatment and ALD functionalization, naturally facilitates scalability. These features allow for a significant increase in throughput as multiple samples can be processed simultaneously without extending the total fabrication time.

Taken together, these results establish AMCA-ALD as a general strategy for engineering ceramic porous channels with independently tunable geometry and surface chemistry, and for disentangling the roles of evaporation, surface energetics, and dynamic wetting in capillary transport. Future work will extend this approach to different template sizes, oxide chemistries, and liquids, while incorporating more detailed, material-specific surface characterization. Hence opening opportunities to tailor fluid transport in printed ceramic architectures for applications in microfluidics, diagnostics, catalysis, and energy systems.

## Supplementary Material



## Notes

Extended experimental setup and extraction details (Figures S1, S3), comprehensive imbibition data sets for all evaluated conditions (Figures S4, S6; Tables S1, S2), static contact angle measurements for each condition (Figures S2, S7, S8), schematic illustrations of localized pore architectures (Figure S5), and a detailed physical breakdown of the imbibition curve-fitting methodology (Section S2) (PDF)

## Abbreviations

AMCA: additive manufacturing combined with colloidal assembly
ALD: atomic layer deposition
ODE: ordinary differential equation
PS: polystyrene

## References

[1] Ishii D., Horiguchi H., Hirai Y., Yabu H., Matsuo Y., Ijiro K., Tsujii K., Shimozawa T., Hariyama T., Shimomura M. (2013). Water transport mechanism
through open capillaries analyzed by direct surface modifications
on biological surfaces. Sci. Rep..

[2] Dudukovic N. A. (2021). Cellular fluidics. Nature.

[3] Mullins B. J., Braddock R. D., Kasper G. (2007). Capillarity
in fibrous filter media:
Relationship to filter properties. Chem. Eng.
Sci..

[4] Zhevago N. K., Glebov V. I. (2007). Hydrogen storage in capillary arrays. Energy Convers. Manage..

[5] Davis M. E. (2002). Ordered
porous materials for emerging applications. Nature.

[6] Ghanbarian B., Hunt A. G., Ewing R. P., Sahimi M. (2013). Tortuosity in Porous
Media: A Critical Review. Soil Sci. Soc. Am.
J..

[7] Gambaryan-Roisman T. (2014). Liquids on
porous layers: wetting, imbibition and transport processes. Curr. Opin. Colloid Interface Sci..

[8] Stone H. A., Stroock A. D., Ajdari A. (2004). Engineering
Flows in Small Devices:
Microfluidics Toward a Lab-on-a-Chip. Annu.
Rev. Fluid. Mech.

[9] Niculescu A.-G., Chircov C., Bîrcă A.
C., Grumezescu A. M. (2021). Fabrication
and Applications of Microfluidic Devices: A Review. Int. J. Mol. Sci..

[10] Stokes K., Clark K., Odetade D., Hardy M., Goldberg
Oppenheimer P. (2023). Advances in lithographic techniques for precision nanostructure
fabrication in biomedical applications. Discover
Nano.

[11] Ohji T., Fukushima M. (2012). Macro-porous ceramics: processing
and properties. Int. Mater. Rev..

[12] Winhard B. F., Haugg S., Blick R., Schneider G. A., Furlan K. P. (2021). Direct writing of colloidal suspensions
onto inclined
surfaces: Optimizing dispense volume for homogeneous structures. J. Colloid Interface Sci..

[13] Winhard B. F., Maragno L. G., Gomez-Gomez A., Katz J., Furlan K. P. (2023). Printing
Crack-Free Microporous Structures by Combining Additive Manufacturing
with Colloidal Assembly. Small Methods.

[14] Li H.-Y., Liu Y.-F., Duan Y., Yang Y.-Q., Lu Y.-N. (2015). Method
for Aluminum Oxide Thin Films Prepared through Low Temperature Atomic
Layer Deposition for Encapsulating Organic Electroluminescent Devices. Materials.

[15] Fujishima A., Zhang X., Tryk D. (2008). TiO2 photocatalysis and related surface
phenomena. Surf. Sci. Rep..

[16] Roach L. (2022). Controlling disorder
in self-assembled colloidal monolayers *via* evaporative
processes. Nanoscale.

[17] Tan A. T. L., Nagelberg S., Chang-Davidson E., Tan J., Yang J. K. W., Kolle M., Hart A. J. (2020). In-Plane Direct-Write
Assembly of Iridescent Colloidal Crystals. Small.

[18] Deegan R. D. (1997). Capillary flow as the cause of ring stains from dried liquid drops. Nature.

[19] Mampallil D., Eral H. B. (2018). A review on suppression and utilization of the coffee-ring
effect. Adv. Colloid Interface Sci..

[20] Hu H., Larson R. G. (2005). Analysis of the
Microfluid Flow in an Evaporating Sessile
Droplet. Langmuir.

[21] Bhardwaj R., Fang X., Somasundaran P., Attinger D. (2010). Self-Assembly of Colloidal
Particles from Evaporating Droplets: Role of DLVO Interactions and
Proposition of a Phase Diagram. Langmuir.

[22] Hu H., Larson R. G. (2002). Evaporation of a
Sessile Droplet on a Substrate. J. Phys. Chem.
B.

[23] Fan F., Stebe K. J. (2004). Assembly of Colloidal
Particles by Evaporation on Surfaces
with Patterned Hydrophobicity. Langmuir.

[24] Li W., Zhang C., Wang Y. (2024). Evaporative
self-assembly in colloidal
droplets: Emergence of ordered structures from complex fluids. Adv. Colloid Interface Sci..

[25] Gruener S., Huber P. (2011). Imbibition in mesoporous silica:
rheological concepts and experiments
on water and a liquid crystal. J. Phys.: Condens.
Matter.

[26] Pan B. (2021). Spontaneous Imbibition
Dynamics of Liquids in Partially-Wet Nanoporous
Media: Experiment and Theory. Transp. Porous
Media.

[27] Huber P., Grüner S., Schäfer C., Knorr K., Kityk A. V. (2007). Rheology
of liquids in nanopores: A study on the capillary rise of water, n-Hexadecane
and n-Tetracosane in mesoporous silica. Eur.
Phys. J. Spec Top.

[28] Pham Q. N., Barako M. T., Tice J., Won Y. (2017). Microscale
Liquid Transport
in Polycrystalline Inverse Opals across Grain Boundaries. Sci. Rep..

[29] Gruener S., Huber P. (2019). Capillarity-Driven
Oil Flow in Nanopores: Darcy Scale Analysis of
Lucas–Washburn Imbibition Dynamics. Transp.
Porous Media.

[30] Lucas R. (1918). Ueber das
Zeitgesetz des kapillaren Aufstiegs von Flüssigkeiten. Kolloid-Z.

[31] Washburn E. W. (1921). The Dynamics
of Capillary Flow. Phys. Rev..

[32] Fries N., Odic K., Conrath M., Dreyer M. (2008). The effect of evaporation
on the wicking of liquids into a metallic weave. J. Colloid Interface Sci..

[33] Sanchez J., Dammann L., Gallardo L., Li Z., Fröba M., Meißner R. H., Stone H. A., Huber P. (2024). Deformation
dynamics of nanopores upon water imbibition. Proc. Natl. Acad. Sci. U. S. A..

[34] Sun Z., Santamarina J. C. (2019). Haines jumps: Pore scale mechanisms. Phys. Rev. E.

[35] Siebold A., Nardin M., Schultz J., Walliser A., Oppliger M. (2000). Effect of
dynamic contact angle on capillary rise phenomena. Colloids Surf. Physicochem. Eng. Asp..

[36] Fries N., Dreyer M. (2008). An analytic solution
of capillary rise restrained by
gravity. J. Colloid Interface Sci..

[37] Hindenlang B., Jimenez A. E., Krekeler T., Ritter M., Diaz A., Holler M., Häntsch Y., Furlan K. P., Zeller-Plumhoff B. (2026). High-resolution analysis
of ordered and disordered isoporous 3D nanostructures
using PXCT. Discovery Nano.

[38] Kim J., Jung Y., Kim H.-Y. (2022). Evaporative
capillary rise. Phys. Rev. Fluids.

[39] Zhang Y., Dong Y. (2026). Interfacial evaporation
and evolution in porous media: a study of
pillar-array micromodel. J. Colloid Interface
Sci..

[40] Radiom M., Chan W. K., Yang C. (2010). Capillary
filling with the effect
of pneumatic pressure of trapped air. Microfluid.
Nanofluid..

[41] Polansky J., Kaya T. (2015). An experimental and numerical study of capillary rise with evaporation. Int. J. Therm. Sci..

[42] Goniakowski J., Finocchi F., Noguera C. (2008). Polarity of
oxide surfaces and nanostructures. Rep. Prog.
Phys..

[43] Mills A., Crow M. (2008). A Study of Factors
that Change the Wettability of Titania Films. Int. J. Photoenergy.

[44] Yang X. M., Zhong Z. W., Diallo E. M., Wang Z. H., Yue W. S. (2014). Silicon
wafer wettability and aging behaviors: Impact on gold thin-film morphology. Mater. Sci. Semicond. Process..

[45] Lee M.-K., Park Y.-C. (2019). Contact Angle Relaxation and Long-Lasting
Hydrophilicity
of Sputtered Anatase TiO_2_ Thin Films by Novel Quantitative
XPS Analysis. Langmuir.

[46] Fu Q., Wagner T., Rühle M. (2006). Hydroxylated
α-Al2O3 (0001)
surfaces and metal/α-Al2O3 (0001) interfaces. Surf. Sci..

[47] Son Y., Lee M.-K., Park Y.-C. (2021). Contact Angle Relaxation on Amorphous,
Mixed-Phase (Anatase + Rutile), and Anatase TiO_2_ Films
and Its Mechanism. Langmuir.

[48] Van
Den Brand J., Van Gils S., Beentjes P. C. J., Terryn H., De Wit J. H. W. (2004). Ageing of aluminium oxide surfaces and their subsequent
reactivity towards bonding with organic functional groups. Appl. Surf. Sci..

[49] Blake T. D., De Coninck J. (2002). The influence of solid–liquid
interactions on
dynamic wetting. Adv. Colloid Interface Sci..

[50] Snoeijer J. H., Andreotti B. (2013). Moving Contact
Lines: Scales, Regimes, and Dynamical
Transitions. Annu. Rev. Fluid. Mech..

[51] Butt H.-J. (2026). Wetting
of granular and porous materials. Adv. Colloid
Interface Sci..

[52] Blake T. D. (2006). The physics
of moving wetting lines. J. Colloid Interface
Sci..

[53] Bonn D., Eggers J., Indekeu J., Meunier J., Rolley E. (2009). Wetting and
spreading. Rev. Mod. Phys..

[54] Blake T. D., Haynes J. M. (1969). Kinetics of displacement. J.
Colloid Interface Sci..

[55] Sedev R. (2015). The molecular-kinetic
approach to wetting dynamics: Achievements and limitations. Adv. Colloid Interface Sci..

[56] Adiga S. P., Zapol P., Curtiss L. A. (2007). Structure
and Morphology of Hydroxylated
Amorphous Alumina Surfaces. J. Phys. Chem. C.

[57] Baek S. (2021). Effects of Tube Radius
and Surface Tension on Capillary Rise Dynamics
of Water/Butanol Mixtures. Appl. Sci..

