# Supplementary material for: Decoupling Geometry and Surface Chemistry in 3D-Printed ALD-Functionalized Porous Ceramic Channels
Source: ACS Omega. 2026 Jun 29;11(27):40232–41. doi: 10.1021/acsomega.6c02566 (PMC13382737; doi:10.1021/acsomega.6c02566)
Supplement: Supplementary file 1 [file ao6c02566_si_001.pdf]

Decoupling geometry and surface chemistry in 3D-printed ALD-functionalised porous ceramic channels

*Antoine E. Jimenez [a], Diego R. Gomes [a], Carina Hedrich [b], Manuel Brinker [c], Fortune Minna [d], Patrick Huber [c], Kaline P. Furlan [a]**

[a] Karlsruhe Institute of Technology, Institute for Applied Materials, Ceramic Materials and Technology, Haid-und-Neu Straße 7, 76131 Karlsruhe, Germany

[b] Electron Microscopy Unit, Institute of Advanced Ceramics, Hamburg University of Technology, Eißendorfer Straße 42, 21073 Hamburg, Germany

[c] Institute for Materials and X-Ray Physics, Hamburg University of Technology, Denickestraße 15, 21073 Hamburg, Germany

[d] Covenant University, KM 10 Idiroko Road, 112104 Ota, Nigeria

1. Experimental methods

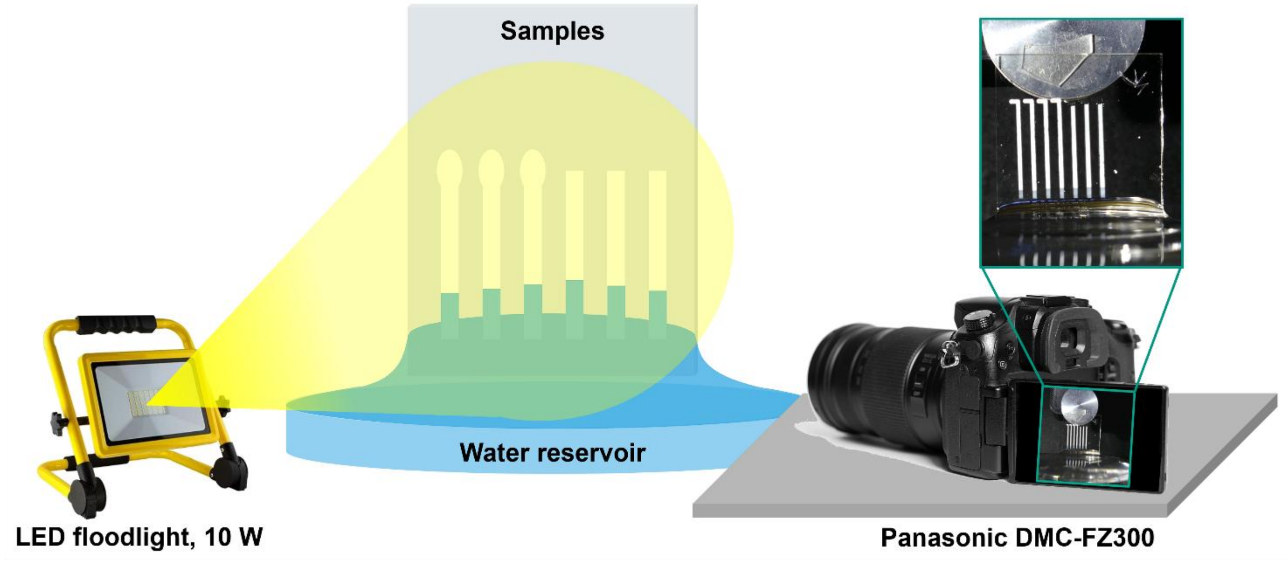

Figure S1. Schematic of the imbibition set-up.

The channels are uniformly in contact with water at the bottom, where the printed lines were cut. The LED floodlight illuminates the porous ceramic channels to enhance the contrast between the white dry sections and the darker imbibed ones. A digital camera captures the water imbibition and saves the resulting videos.

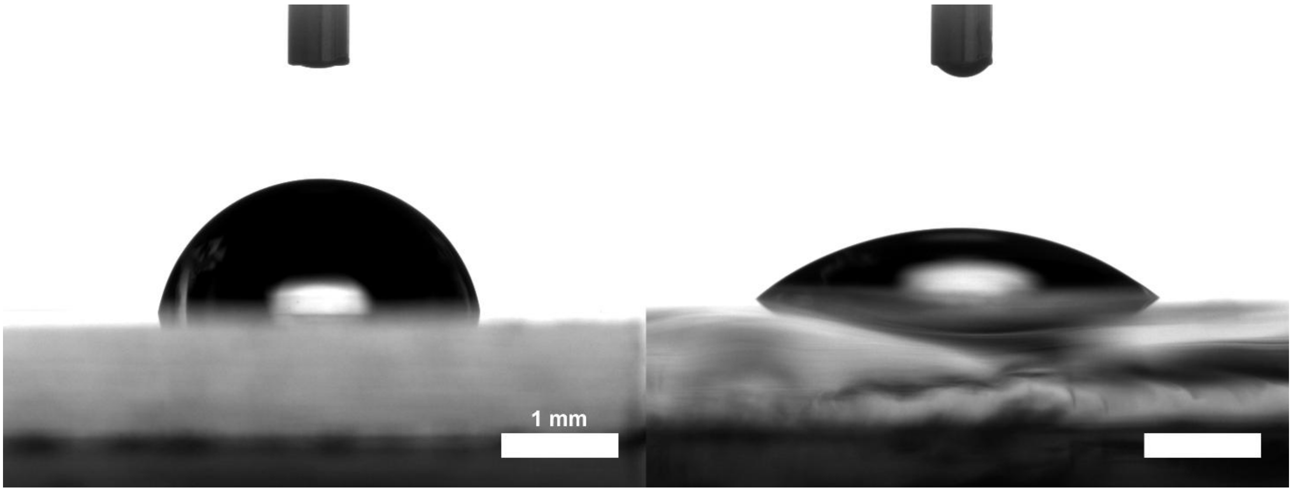

Figure S2. Contact angle measurements on Al_2O_3 (left) and TiO_2 (right) ALD-coated substrates in before burn-out conditions. Results are $\theta_{alumina}$ = 80.54 ° and $\theta_{titania}$ = 38.98 °, respectively.

A 3 μL Milli-Q water droplet is deposited on each substrate, next to the printed lines, without touching them. The contact angles are measured directly in the software provided by the equipment.

2. Results & extended Analysis

   a. Capillary rise in the Lucas–Washburn regime

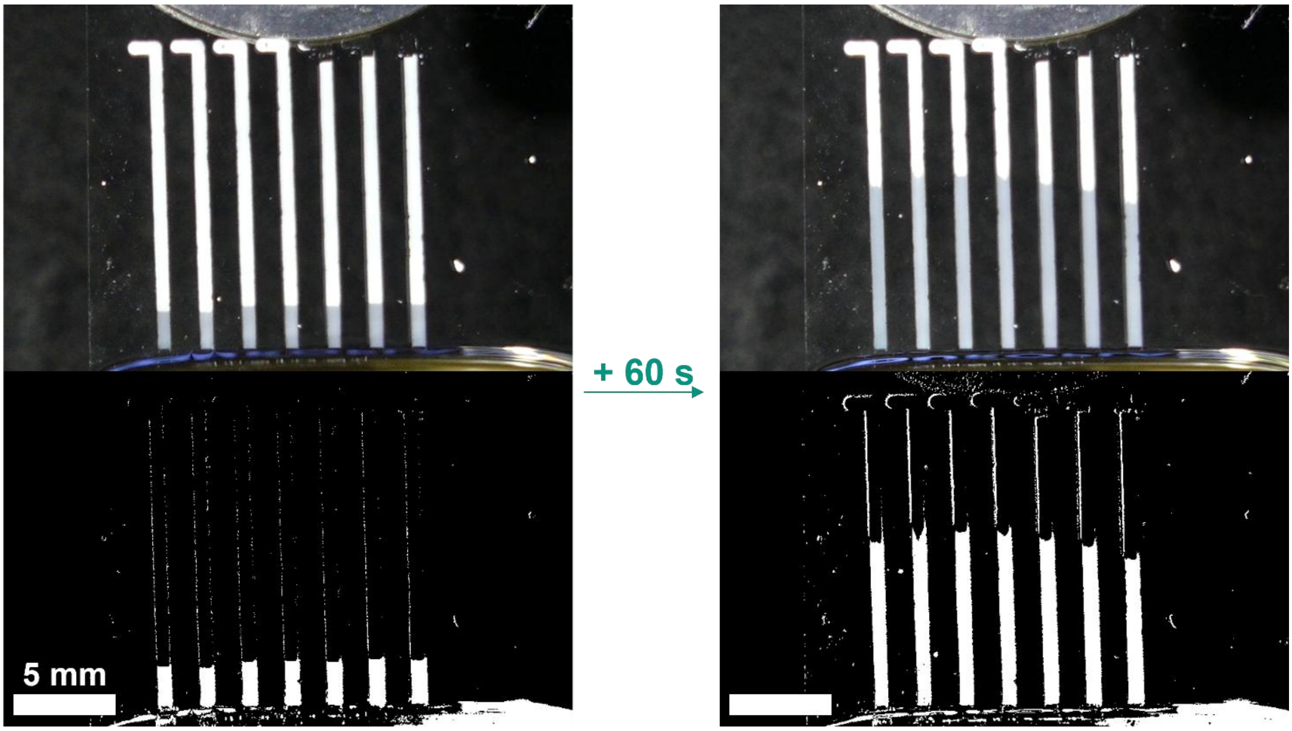

Figure S3. Evolution of the menisci shape during spontaneous imbibition: (left) early rapid rise regime with flat menisci and (right) later resistance-limited regime exhibiting concave menisci.

During the initial rapid imbibition regime, the menisci rise with a nearly flat profile across the channels' widths, indicating a consistent rising rate throughout the porous cross-section. In contrast, during the resistance-limited regime, the menisci gradually deform into a concave shape, with the water front rising higher near the channel's edges. This transition reflects the increasing influence of interfacial viscous resistance and evaporation, which cause local fluctuations in the local imbibition rate.

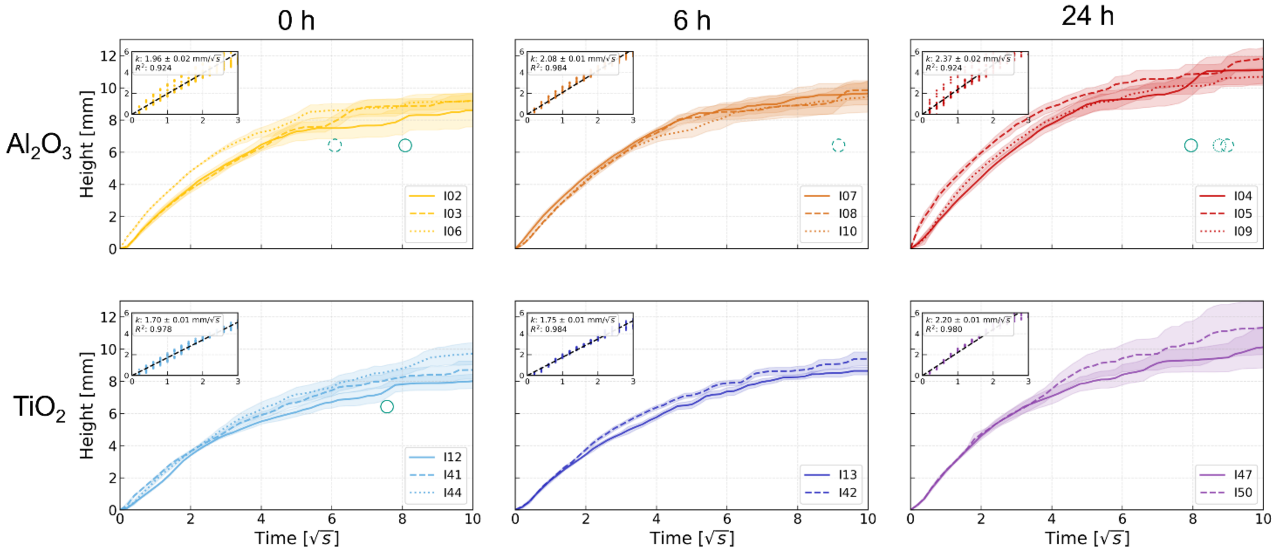

Figure S4. Imbibition height as a function of the square root of time for Al_2O_3 and TiO_2 channels at different after-burn-out times with a focus on the Lucas–Washburn regime. Lines represent the mean values, while the shaded areas indicate the standard deviation (± 1 SD) and circles mark Haines jumps.

The imbibition height h plotted against $\sqrt{t}$ for all channels and conditions is presented in Figure S5. For each sample, capillary rise at an early stage is well described by a linear fit of the form $h(t) = S\sqrt{t}$, where S is the Lucas-Washburn slope. In the ideal Lucas-Washburn description, the imbibition height obeys the equation of the same name (Equation S1)[1,2].

Equation S1.

$$h\,(t) = \sqrt{\frac{\sigma \cdot r \cdot cos\,\theta}{2\mu} t}$$

The Lucas-Washburn equation relates the surface tension σ [N·m^{-1}], the viscosity μ [Pa·s = N·m^{-2}·s], the (effective) static contact angle θ and the Lucas-Washburn equivalent pore radius r [m]. Thus, the experimentally determined slope S can be interpreted as $S = \sqrt{\frac{\sigma \cdot r \cdot \cos(\theta)}{2\mu}}$, and is used as input for the subsequent Siebold and Fries-Dreyer analysis[3,4]. The linearity of the plots at the beginning confirms that the early imbibition regime is dominated by capillary forces and obeys the Lucas–Washburn scaling. The extracted slopes and the associated final heights reached after 100 seconds

(H100) and associated standard deviations (± 1 SD) are reported in Table S1. The parameter extraction is described in further sections (Sections S2.(b, c)).

material	time after burn-out (h)	cut	H100 (mm)	SD (mm)	slope ($mm \cdot s^{-1/2}$)	SD ($mm \cdot s^{-1/2}$)
Al_2O_3	0	1	9.27	0.190	2.02	0.192
Al_2O_3	0	2	8.71	0.570	1.94	0.246
Al_2O_3	6	1	10.2	0.0478	2.06	0.0404
Al_2O_3	6	2	9.16	0.251	2.10	0.0808
Al_2O_3	24	1	11.1	0.146	2.25	0.161
Al_2O_3	24	2	11.1	0.495	2.48	0.270
TiO_2	0	1	8.79	0.684	1.73	0.0926
TiO_2	0	2	8.83	0.619	1.69	0.0389
TiO_2	6	1	9.13	0.476	1.77	0.0983
TiO_2	6	2	8.95	0.307	1.75	0.0614
TiO_2	24	1	9.31	0.288	2.12	0.0262
TiO_2	24	2	11.9	0.632	2.27	0.0126
Al_2O_3	all	1	9.99	0.766	2.20	0.189
Al_2O_3	all	2	9.57	1.23	2.19	0.343
TiO_2	all	1	9.04	1.58	1.85	0.278
TiO_2	all	2	9.73	0.439	1.87	0.188

Table S1. Imbibition results according to the materials, after-burn-out times and cuts. H100 represents the imbibition height after 100 seconds and SD the standard deviation (± 1 SD).

b. Geometrical determination of pore-scale radii

i. Pore throats at former templating sphere-sphere contacts

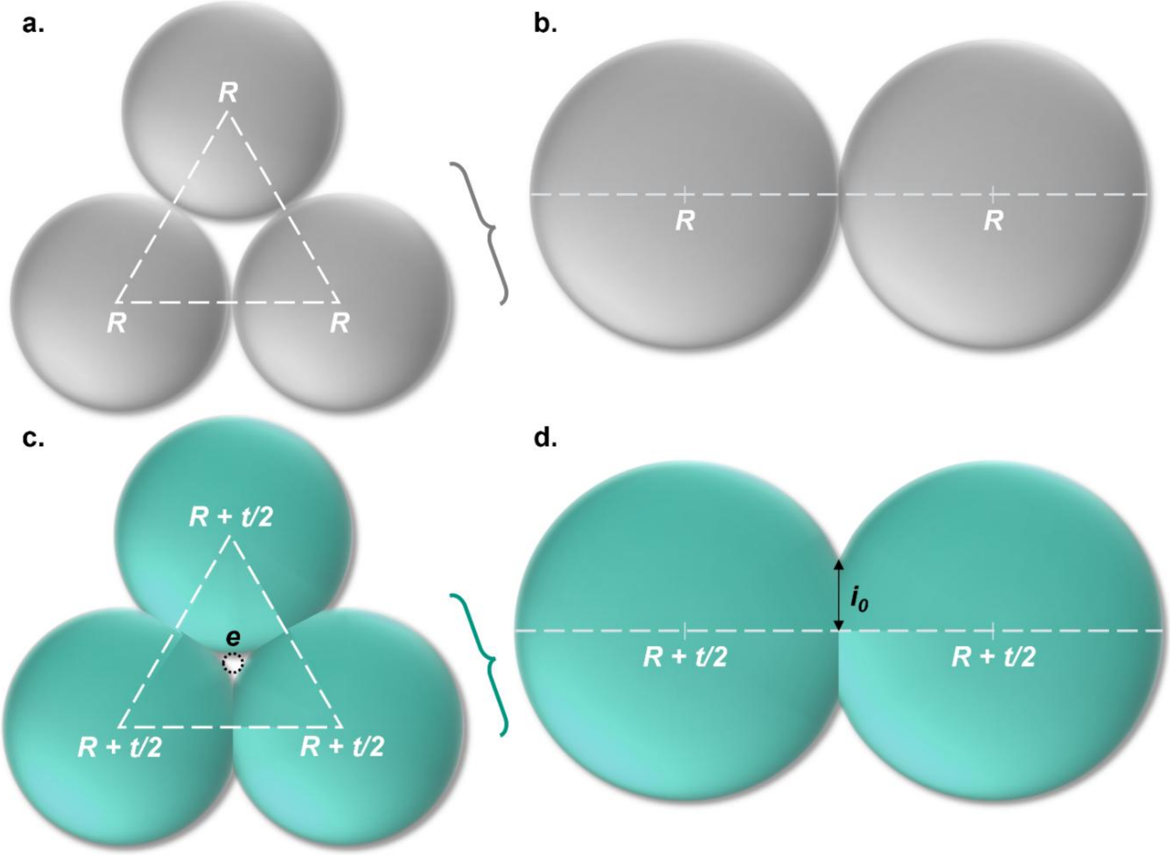

Figure S5. Geometrical determination of the static radius R_S. (a,b) Polymeric templates in contact prior to coating. (c,d) Corresponding pores after ALD coating and template burn-out, showing (c) the pore junction throat radius e and (d) the internal pore throat radius i_0.

The pore network arises from a random packing of spherical templating polystyrene particles with radius R, conformally coated by an ALD film of thickness t and subsequently burnt out in air. After burn-out, the ceramic shells form pores in contact where the original template touched. As a result, each pore is connected to several neighbouring pores through pore throats, with the connectivity determined by the local coordination number of the initial random (Figure 3(c))[5].

To estimate the characteristic size of these throats, consider two identical coated spheres of radius $R+t$ whose centres are separated by a distance of $2R$, corresponding to initial tangential contact of the uncoated templates. The outer surfaces of the coated spheres intersect along a circle. Neglecting fracture details, the radius of that circle provides an upper bound for the internal pore throat radius $i_0 = \sqrt{2R \cdot t + t^2}$. For the present structures, with template diameter $D = 2R = 2.94$ µm and ALD

thickness t = 65 nm, this expression yields $i_0 \approx 0.44$ µm. In practice, the pore throat radius i_0 is expected to be smaller than this ideal value, but remains of the same order of magnitude. In the present disordered porous channel, each pore is connected to numerous neighbouring pores through multiple such internal throats, with a coordination number that varies locally due to the stochastic nature of packing[5].

ii. External three-sphere junction throats

In addition to internal pore throats, free spaces exist in the interstitial regions where three or more templating spheres meet. The narrowest constrictions in these interstitial voids occur at three-sphere junctions. In the plane passing through the centres of three equal templating spheres, these spheres form a triangle, and the gap between them defines a curved triangular opening. From elementary geometric considerations, the radius of the largest inscribed circle in this resulting junction throat is given by $r_{tri} = R\left(\frac{2}{\sqrt{3}} - 1\right) \approx 0.155R$. After ALD, the effective radius of this external junction throat is reduced by the coating thickness t, yielding an approximate radius $e \approx r_{tri} - t \approx 0.155R - t$. For R = 1.47 µm and t = 65 nm, this gives $e \approx 0.16$ µm, significantly smaller than the internal pore throat radius i_0.

iii. Identification of the relevant static radius for capillary rise

Capillary rise in a disordered porous network is governed not by the size of the pore bodies but by the smallest constrictions along percolating flow paths. This follows directly from the Laplace pressure, as $\Delta P = 2\gamma \cdot \cos\theta / \mathrm{r_{throat}}$. Consequently, the narrowest pore throats dominate the capillary driving force and control the advance of the meniscus.

Although the spherical pores are large and well connected via relatively wide pore throats (i), fluid transport is ultimately limited by narrower constrictions formed at junctions between templating

spheres, with the smallest being the three-sphere junction throat (e). These constrictions act as capillary bottlenecks that must be traversed by the advancing liquid front. Importantly, the smallest constrictions simultaneously provide the capillary pressure required for imbibition while acting as kinetic bottlenecks that limits the rising rate of the liquid front.

Independent 3D tomography of similar ALD-derived porous ceramics prepared from ~1.48 µm templates and ~60 nm ALD thickness shows a bimodal distribution of pore throat diameters[5]. One population is centred around ~100 nm, while a second population appears near ~300 nm. The smaller mode agrees with the geometrical estimate for the external junction throat size e, whereas the larger mode corresponds approximately to half of the internal pore throat upper bound i_0. This confirms the existence of two distinct and abundant classes of constrictions within a porous channel.

On this basis, when introducing a static pore radius R_S into the capillary term of the wicking model, the relevant length scale is identified as the external junction radius $R_s \equiv e \approx 0.155R - t$, which captures the capillary pressure associated with the tightest throat that the meniscus must traverse.

iv. Hydrodynamic radius and permeability estimation

The Siebold analysis distinguishes between the static radius R_S and a hydrodynamic radius R_D, which characterises viscous conductance. The ratio $r = \frac{R_D^2}{R_S}$ is the Washburn radius, obtained from the initial slope of $h^2(t)$ in the Lucas–Washburn regime using $\sigma = 72.8 \cdot 10^{-3}$ N·m^{-1}, $\mu = 10^{-3}$ Pa·s, and the corresponding static contact angles. With r and R_S known, the hydrodynamic radius follows as $R_D = \sqrt{r \cdot R_S}$.

Assuming Hagen–Poiseuille-like flow through an assembly of capillaries, the ratio of porosity ϕ to permeability K is given by Siebold et al.[3], $\frac{8}{R_D^2} = \frac{\phi}{K}$. This relation is used to evaluate the intrinsic permeability K from the experimentally accessible parameters.

c. Modelling of imbibition dynamics

i. Governing equation: Siebold and Fries-Dreyer framework

The macroscopic evolution of the imbibition height *h(t)* is described using the Fries–Dreyer model, as introduced in the main text (Section 3.b). This framework extends the classical Lucas–Washburn description by explicitly accounting for gravity and evaporation. The governing ordinary differential equation (ODE) states:

Equation 1.

$$\frac{dh}{dt} = \frac{a}{h} - b - c \cdot h$$

where the three terms represent, respectively, the capillary driving force, the gravitational resistance and evaporative losses.

The coefficients are defined as $a = \frac{2\sigma cos\theta}{\phi\mu}\frac{K}{R_s}$, $b = \frac{\rho g K}{\phi\mu}$, $c = \frac{\dot{m}_e(W+T)}{\rho W T \phi}$, here σ is the surface tension [$N \cdot m^{-1}$], μ the viscosity [$Pa \cdot s = N \cdot m^{-2} \cdot s$], ρ the liquid density [$kg \cdot m^{-3}$], g the gravitational acceleration [$m \cdot s^{-2}$], K the intrinsic permeability [m^2], ϕ the porosity, R_s the static radius of the pore throats [m]. The parameter $\dot{m}_e$ is the evaporative mass flux [$kg \cdot m^{-2} \cdot s^{-1}$] related to the macroscopic channel dimensions W (width) and T (thickness or height). The coefficients have units/dimensions a [$m^2 \cdot s^{-1}$], b [$m \cdot s^{-1}$] and c [s^{-1}].

ii. Parameter extraction and fitting procedure (coefficients a, b, and c)

The model coefficients and fitting procedure were calculated using respectively $\sigma = 0.0728$ $N \cdot m^{-1}$ and $\mu = 0.001$ Pa·s as the surface tension and viscosity of water, with a density $\rho = 998$ $kg \cdot m^{-3}$ and gravitational acceleration $g = 9.81$ $m \cdot s^{-2}$. A contact angle $\theta = 0$ ° was used for all samples but for Al_2O_3 ones tested 24 hours after burn-out, for which $\theta = 11$ °. The average channel width and height of $W = 600$ µm and $T = 15$ µm. A porosity of $\phi = 0.8$ was estimated based on structural analysis[5]. The characteristic static pore radius governing capillary rise was identified as the smaller junction throat size, $Rs \equiv e = 0.16$ µm.

- *Coefficient a*

During the early stage of imbibition, gravitational and evaporative contributions are negligible. As a result, Equation 1 reduces to $\frac{dh}{dt} \approx \frac{a}{h}$. Multiplying by $2h$ and integrating yields $\frac{d(h)^2}{dt} \approx 2a$. Therefore, the parameter a can be directly obtained from the slope of a linear fit to $h^2(t)$ in the early-time regime (Figure S5).

- *Coefficient b*

The coefficient b captures the gravitational resistance to imbibition. It is determined independently without fitting, by calculating the permeability K using the hydrodynamic radius R_D from Siebold's relation (Section S2.b.iv). Substituting this value of K into the expression for b yields the gravitational contribution to the dynamics.

- *Coefficient c*

At long times, the imbibition height approaches a steady-state value h_∞, such that $dh/dt \to 0$. Under this condition, Equation 1 becomes $0 = \frac{a}{h_\infty} - b - ch_\infty$. Solving for c gives $c = \frac{a - bh_\infty}{h_\infty^2}$. The evaporative mass flux $\dot{m}_e$ is then obtained by inverting the expression for c: $\dot{m}_e = c\,\frac{\rho W T \phi}{W+T}$. Sample-specific values of h_∞, c, and $\dot{m}_e$ are reported in Table S2.

- *Uncertainty analysis*

Both coefficients a and b are calculated from the Lucas-Washburn slope, the corresponding relative slope errors are calculated to be 2.2% and 5.3%, respectively. As the coefficient c is solved based on both previous coefficients and the steady-state value h_∞, the error is found to be greater with 9.3%.

material	time after burn-out (h)	cut	h_∞ (mm)	c (s^{-1})	$\dot{m}_e$ ($g \cdot m^{-2} \cdot s^{-1}$)
Al_2O_3	0	1	9.27	0.0270	0.316
Al_2O_3	0	2	8.71	0.0287	0.336
Al_2O_3	6	1	10.17	0.0224	0.262
Al_2O_3	6	2	9.16	0.0291	0.340

Al_2O_3	24	1	11.1	0.0230	0.269
Al_2O_3	24	2	11.1	0.0284	0.332
TiO_2	0	1	8.79	0.0214	0.251
TiO_2	0	2	8.83	0.0203	0.237
TiO_2	6	1	9.13	0.0198	0.232
TiO_2	6	2	8.95	0.0205	0.239
TiO_2	24	1	9.31	0.0301	0.352
TiO_2	24	2	10.7	0.0209	0.244

Table S2. Imbibition fitting and evaporation parameters according to the materials, after-burn-out times and cuts.

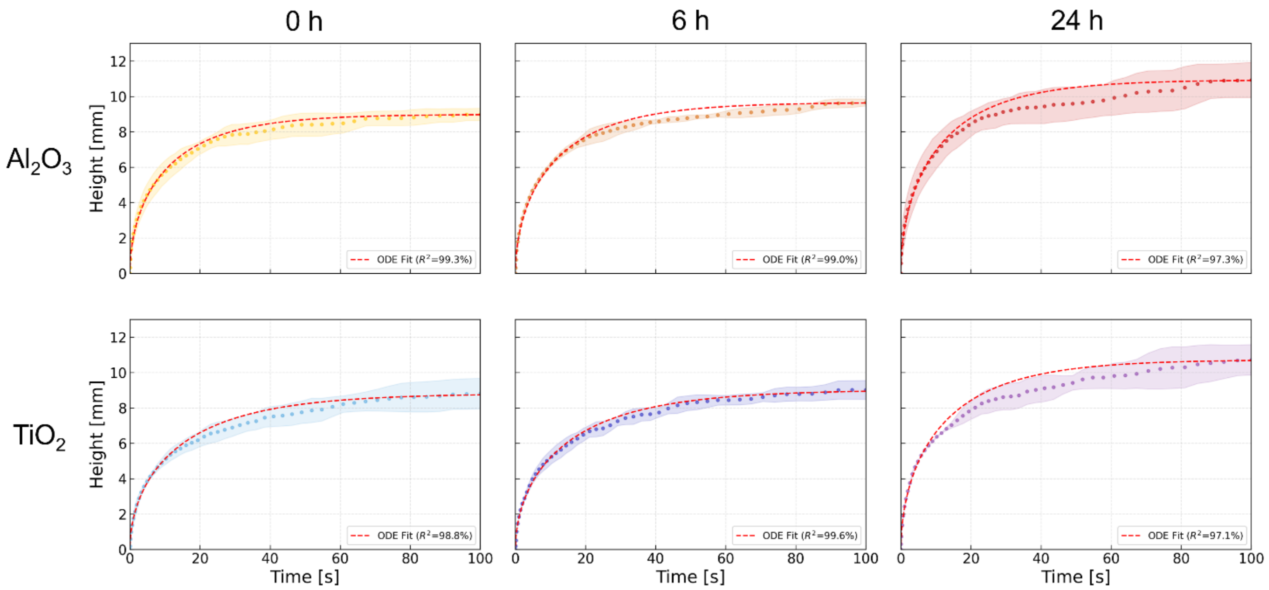

Figure S6. Model fits for all materials and after-burn-out times. Lines represent the mean values, while the shaded areas indicate the standard deviation (± 1 SD) of the experimental data.

d. Surface chemistry evolution

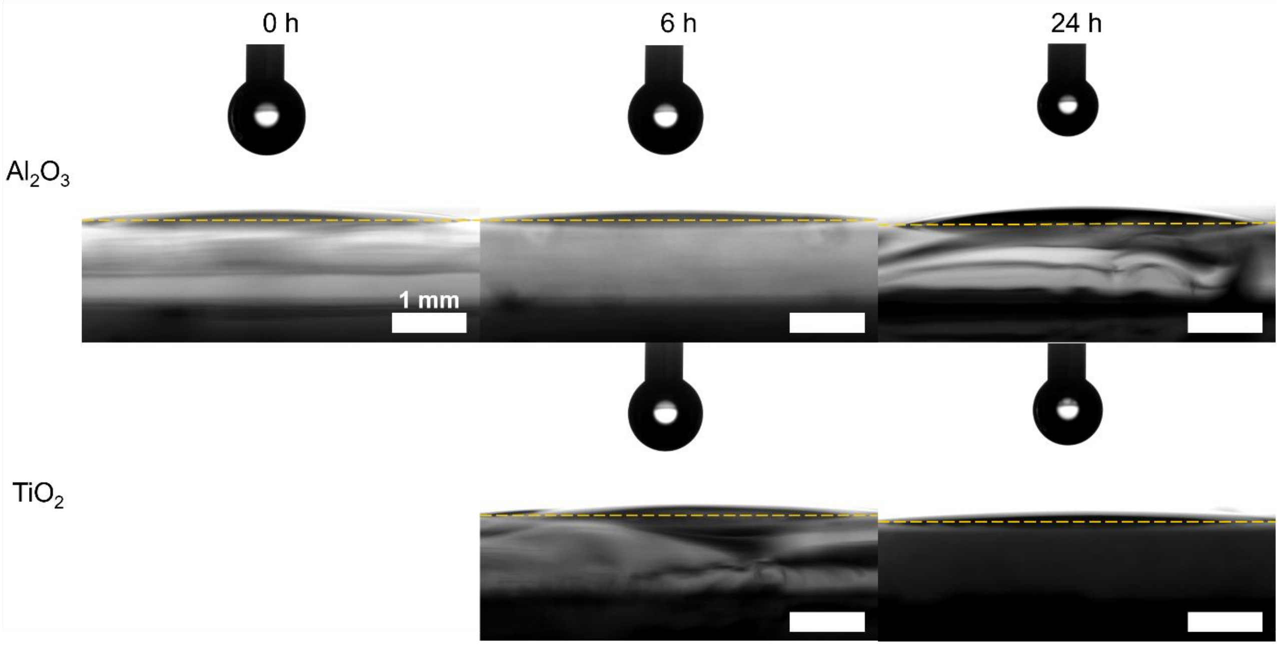

Figure S7. Contact angle measurements on Al_2O_3 (top) and TiO_2 (bottom) ALD-coated substrates at different times after template burn-out (0, 6 and 24 h). Both oxides exhibit superhydrophilic behaviour ($\theta \approx 0$ °). Partial hydrophobic recovery is observed only for Al_2O_3 after 24 h, $\theta \approx 11 \pm 1$ °.

A 3 µL Milli-Q water droplet is deposited on each substrate, next to the printed lines, without touching them. The contact angles are measured directly in the software provided by the equipment. Immediately after burn-out, water droplets spread completely on both Al_2O_3 and TiO_2 surfaces, indicating a superhydrophilic state. Over time, a gradual relaxation of wettability is observed with the Al_2O_3 channels after 24 hours exhibiting partial recovery with a measurable contact angle ($\theta = 11 \pm 1$ °), whereas TiO_2 remains superhydrophilic within the experimental timeframe.

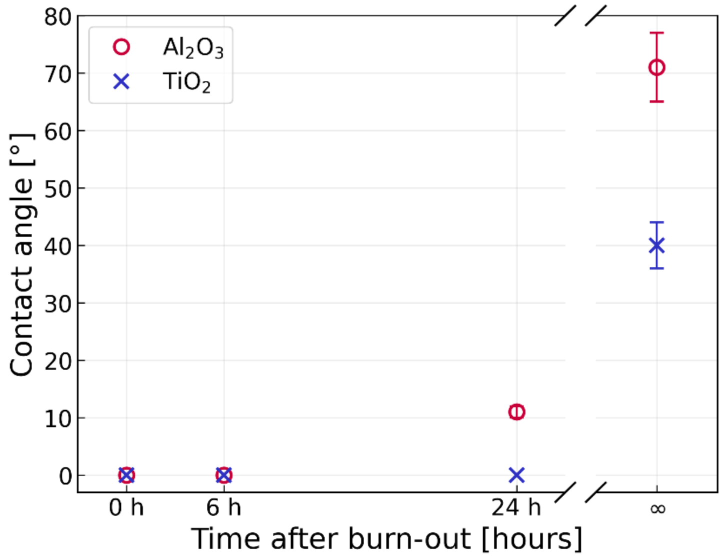

Figure S8. Contact angle measurements on flat Al_2O_3 and TiO_2 ALD-coated substrates at different times after template burn-out (0, 6, 24 h and "infinite" time) with respective error bars.

REFERENCES

1. Lucas, R. Ueber das Zeitgesetz des kapillaren Aufstiegs von Flüssigkeiten. *Kolloid-Z.* **23**, 15–22 (1918).

2. Washburn, E. W. The Dynamics of Capillary Flow. *Phys. Rev.* **17**, 273–283 (1921).

3. Siebold, A., Nardin, M., Schultz, J., Walliser, A. & Oppliger, M. Effect of dynamic contact angle on capillary rise phenomena. *Colloids Surf. Physicochem. Eng. Asp.* **161**, 81–87 (2000).

4. Fries, N. & Dreyer, M. An analytic solution of capillary rise restrained by gravity. *J. Colloid Interface Sci.* **320**, 259–263 (2008).

5. Hindenlang, B. *et al.* High-resolution analysis of ordered and disordered isoporous 3D nanostructures using PXCT. *Discov. Nano* **21**, 46 (2026).
